# Predictive Optimization of Vibrations in a Biodiesel-Fueled Variable Compression Ratio Engine Using BO-CNN-LSTM

**DOI:** 10.1021/acsomega.6c08418

**Published:** 2026-10-16

**Authors:** Ihsan Uluocak

**Affiliations:** Department of Mechanical Engineering, Ceyhan Engineering Faculty, 37506 Cukurova University, Adana 01950, Turkiye

## Abstract

Integrating sustainable biodiesel blends into variable compression ratio (VCR) diesel engines significantly alters combustion dynamics and structural vibrations. The present study introduces a Bayesian optimized 1D convolutional neural network-long-short-term memory (CNN-LSTM) deep learning framework designed for optimizing the multiaxis engine vibrations. The predictive architecture processes a six-parameter input matrix encompassing engine load, compression ratio (CR), and critical thermophysical fuel properties. A precise evaluation demonstrated the superior fidelity of the hybrid network, achieving up to a coefficient of determination (*R* ^2^) of 0.985 and a root mean square error (RMSE) of 0.288 m/s^2^, substantially outperforming optimized support vector machine (SVM) and multilayer perceptron (MLP) benchmarks. Multiaxis spatial stability was visually confirmed using Taylor diagrams, while a 3000-iteration bootstrap analysis validated algorithmic robustness within a narrow 95% confidence interval (0.38–0.79 m/s^2^). Permutation feature importance and SHapley Additive exPlanations (SHAP) analysis clarified the thermodynamic decision-making process, revealing that elevated engine load and CR intensify mechanical ringing, whereas the higher density and viscosity of biodiesel blends act as natural vibration dampeners. High-resolution contour maps generated through systematic response surface mapping provided precise predictive optimization surfaces. Deploying this advanced methodology establishes a highly reliable, cost-effective virtual calibration tool for next-generation biodiesel-fueled engines, eliminating the necessity for exhaustive physical testing.

## Graphical abstract

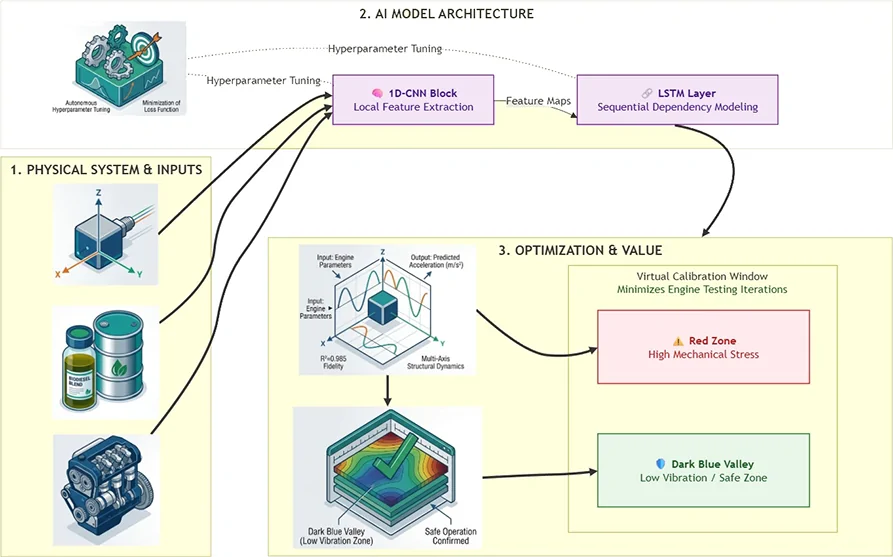

## 1 Introduction

The rapid development of the global economy relies heavily on the consumption of fossil fuels, which has led to severe energy shortages and environmental degradation.^1^ Internal combustion engines (ICEs) remain a primary power source across the transportation and agricultural sectors due to their high thermal efficiency.^1,2^ However, compression ignition (CI) engines consume vast amounts of petroleum and emit significant pollutants, prompting strict international emission regulations.^1^ For the purpose of overcoming the environmental and health impacts of traditional fossil fuels, researchers are increasingly focused on low-carbon and renewable alternative fuels.^1,3^ Among these, biodiesel has emerged as a highly viable and sustainable alternative that can be utilized in existing diesel engines to mitigate greenhouse gas emissions and reduce the carbon footprint of the transportation sector.^4^

When transitioning to alternative fuels like biodiesel, optimizing engine operational parameters is essential to maintain or improve performance. The compression ratio (CR) is one of the most critical design parameters in an engine’s thermodynamic cycle.^5^ Increasing the CR generally leads to higher temperatures at the end of the compression stroke, promoting better fuel combustion and improving energy efficiency.^6^ However, altering the CR and introducing different biodiesel blends significantly impacts the in-cylinder pressure development and the heat release rate.

These variations in combustion dynamics govern the cyclic excitation forces transmitted to the crankshaft and the overall engine structure, directly influencing the engine’s noise, vibration, and harshness (NVH) characteristics.^4,7^ Engine vibration analysis provides critical insights into the mechanical health, operational efficiency, and structural integrity of the system.^8,9^ Excessive vibration can lead to mechanical fatigue, increased wear, and reduced component life.^10,11^ Therefore, measuring multiaxis (3-axis) vibration is a vital diagnostic tool to understand the complex mechanical responses of a diesel engine operating under varying compression ratios and diverse biodiesel blends.

Predicting the complex thermodynamic and mechanical behaviors of internal combustion engines such as vibration and performance, using traditional empirical or three-dimensional computational fluid dynamics (CFD) models is computationally expensive and frequently falls short in capturing highly nonlinear dynamics across varied operating conditions.^12,13^ Consequently, data-driven surrogate models powered by machine learning (ML) and deep learning (DL) have become the state-of-the-art approach for engine modeling and real-time optimization.^11,14^

Traditional machine learning algorithms, such as SVM and MLP, have proven highly effective in mapping the nonlinear relationships between engine inputs (e.g., fuel type, CR) and outputs, offering rapid evaluation times and robust predictive accuracy.^2^ Furthermore, for complex, sequential data such as vibration signals, advanced deep learning architectures are required.^15^ CNNs are exceptionally capable of extracting spatial features from vibration signals, while LSTM networks excel at processing temporal dependencies and capturing the cycle-to-cycle “memory” of engine dynamics without suffering from the vanishing gradient problems of standard recurrent networks.^11,16^ A hybrid CNN-LSTM architecture leverages both spatial feature extraction and sequential learning, providing a highly comprehensive representation of dynamic mechanical faults and combustion characteristics.^17^

### 1.1 Research Gap and Proposed Contribution

Existing literature has extensively evaluated the isolated effects of biodiesel blends or compression ratios on basic engine performance.^4–6^ However, a distinct lack of comprehensive studies integrating multiaxis vibration analysis with advanced artificial intelligence-based predictive modeling persists. The complex, nonlinear trade-offs between varying biodiesel thermophysical properties, variable compression ratios, and the resulting multiaxis mechanical vibrations remain inadequately mapped by modern deep learning frameworks. To clearly delineate the boundaries of current research and highlight the specific advancements achieved in this study, a comprehensive comparison with recent engine modeling and vibration prediction literature is presented in Table 1.

**Table 1:** Literature Review for Proposed System

reference	applied algorithm	target application & fuel type	predictive targets	explainability (XAI)/interpretability	optimization/virtual calibration
Mehnatkesh et al.^2^	temporal KAN	hydrogen/diesel biodiesel-fueled engine	engine performance and emissions	no	no
Zhang et al.^10^	ML-based inversion	general internal combustion engine	crankshaft stress and general vibration	no	no
Tang^11^	LSTM-GAN	turbofan engines	remaining useful life (RUL) prediction via Vibration	no	no
Huang and Xu^15^	CWT-CNN	general engineering structures	vibration pattern recognition	no	no
Dao et al.^17^	BO-CNN-LSTM	hydro-turbine systems	mechanical fault diagnosis	no	no
Das et al.^19^	optimized CatBoost	medical/general classification	classification targets	yes (SHAP)	no
proposed study	BO-CNN-LSTM	VCR diesel engine (biodiesel blends)	multiaxis (*X*, *Y*, *Z*) structural vibration	yes (SHAP & permutation feature importance)	yes (Monte Carlo optimization surfaces)

Addressing this critical research gap, this study systematically investigates the coupled impacts of diverse biodiesel blends and variable compression ratios on the 3-axis vibration characteristics of a diesel engine. Furthermore, a highly robust digital framework is proposed for the accurate forecasting of these complex mechanical responses. The primary novelties and scientific contributions of this research are outlined as follows:

- Advanced hybrid architecture: developing a highly accurate BO-CNN-LSTM deep learning architecture specifically designed to forecast complex multiaxis (*X*, *Y*, and *Z*) structural vibrations based on direct physical and operational input parameters.
- Model generalization: establishing and proving strong predictive generalization capabilities across a highly diverse, multidimensional matrix of variable compression ratios and biodiesel blends. This ensures reliable interpolation across untested different biodiesel types and different fuels with additives.
- Rigorous algorithmic benchmarking: conducting an extensive comparative evaluation of the proposed deep learning framework against optimized traditional data-driven modeling approaches, specifically SVM and MLP algorithms.
- Explainable AI: deploying SHAP and feature importance analysis to physically interpret the thermodynamic impact of individual fuel properties and compression ratios, alongside generating continuous systematic response surface mapping optimization surfaces for the virtual calibration and operational optimization of biodiesel-fueled engines.

The findings of this study establish a robust, high-fidelity digital framework essential for the continuous condition monitoring and predictive optimization of modern alternative-fuel internal combustion engines.

## 2 Methodology

### 2.1 Data Acquisition and Preprocessing Strategy

The experimental data for this study was collected utilizing an internal combustion engine testbed, designated on Automotive engineering laboratory of Cukurova University. The core of this setup features a Kirloskar Oil Engine-240, which is a highly adaptable variable compression ratio (VCR) diesel engine. The details of the engine is listed on Table 2.

**Table 2:** Parameters of Kirloskar Oil Engine-240

parameter	specification
brand/model	Kirloskar Oil Engines/240
configuration	single cylinder, four stroke, water cooled
combustion principle	compression ignition (direct injection)
displacement	661 cc
bore × stroke	87.5 mm × 110 mm
operating speed (Min/Max)	1200/2000 rpm
rated power	3.5 kW@1500 rpm
compression ratio (CR)	12:1 to 18:1 (variable)
peak cylinder pressure	77.5 kg/cm^2^
total weight	160 kg

A Triaxial ICP accelerometer sensor (PCB Piezotronics, model 356A33) was rigidly mounted on the engine structure for precise structural response monitoring for 3 axes.^18^ The details of the sensor can be seen on Table 3. The dynamometer is an eddy current dynamometer which is designed to fix the engine speed on 1500 rpm.

**Table 3:** Specifications of Accelerometer

measurement range	±4905 m/s^2^ pk
sensitivity (±10%)	1.02 mV/(m/s^2^)
frequency range (±5%) (x axis)	2 to 7000 Hz
frequency range (±5%) (*y* or z axis)	2 to 10,000 Hz
resonant frequency	≥55 kHz
temperature range (operating)	–54 to+121 °C
sensing geometry	shear
sensing element	ceramic

The raw analog vibration signals were acquired and digitized through a Soundbook universal portable measuring system, operating in conjunction with a Panasonic Toughbook CF-19 personal computer. Signal processing and real-time data visualization were managed using SAMURAI v2.6 software from SINUS Messtechnik GmbH. This advanced software environment is capable of performing single or double mathematical integration of the time-domain acceleration signals, allowing for the precise derivation of velocity and displacement metrics. Furthermore, the measurement suite supports strict signal filtering in accordance with ISO 10816, ISO 7919, and ISO 2954 international standards, effectively capturing a broad and accurate vibration frequency range from 2 Hz up to 20 kHz. The thermophysical properties of the tested fuels were experimentally measured at the Automotive Engineering Laboratories of Cukurova University in accordance with international standards. Specifically, fuel density was measured using a Kyoto Electronics DA-130 densimeter (ASTM D4052), kinematic viscosity with a Tanaka AKV-202 automated system (ASTM D445), and gross heating value via an IKA-Werke C2000 bomb calorimeter (ASTM D240). Additionally, the cetane number was determined utilizing a Zeltex ZX440 near-infrared analyzer.

The experimental matrix was systematically constructed by fuelling the engine with sixteen distinct fuel configurations throughout the testing phase. The baseline pure low-sulfur diesel is denoted as D100. Three distinct biodiesel types, Sunflower (SB), Canola (CaB), and Corn (CoB) were blended with diesel at volumetric ratios of 20%, 40%, 60%, 80%, and 100%. The specific nomenclature adopted for these blends indicates the biodiesel type and its volumetric percentage. For instance, SB20 represents a blend of 20% sunflower biodiesel and 80% diesel, while CaB100 denotes 100% pure canola biodiesel. The same naming convention is systematically applied across all sunflower (SB20–SB100), canola (CaB20–CaB100), and corn (CoB20–CoB100) configurations. The resulting comprehensive data set comprises 96 distinct observational samples, capturing the mechanical response across these diverse fuel blends under different operational parameters. The predictive input matrix is constructed from six critical, direct physical properties encompassing both the engine operating conditions and the thermophysical fuel characteristics: compression ratio (CR), engine load (kW), fuel density (kg/m^3^), kinematic viscosity (mm^2^/s), cetane number, and gross heating value (MJ/kg) which are prepared and tested on Automotive Engineering Laboratories of the Çukurova University. Each of the 16 fuel configurations was systematically tested under three distinct compression ratios (12, 14, and 16) and two specific engine loads (0 kW and 2 kW). This combinatorial matrix (16 fuels × 3 CRs × 2 loads) generated exactly 96 steady-state observational samples. At each of these 96 operating conditions, the engine was allowed to reach thermal and mechanical stability before the multiaxis vibration signals were recorded. The target variable mapped against these inputs is the corresponding engine vibration magnitude (vibration *X*, *Y* and *Z*, measured in m/s^2^).

Standard *Z*-score normalization was systematically applied to all input variables and the target output prior to model training. This essential mathematical transformation scales the data set to a mean of zero and a standard deviation of one, preventing features with inherently larger numerical magnitudes (e.g., gross heating value) from disproportionately dominating the learning algorithm. Furthermore, since the data set is not big, a 5-fold cross-validation (CV) strategy was incorporated into the architectural training phase.^19^ This partitioning methodology objectively evaluates the model’s generalization capability across unseen data subsets, mitigating the risk of overfitting and ensuring that the reported performance metrics reflect true predictive reliability rather than mere memorization of the experimental data. In each of the five iterations, four subsets (80% of the data) were utilized for training the algorithm, while the remaining single subset (20%) served as the unseen testing set. The term “testing set” throughout this study strictly refers to these out-of-fold validation subsets. The final statistical performance metrics and visual diagnostic tools represent the aggregated predictions across all five independent testing sets.

### 2.2 Proposed Hybrid BO-CNN-LSTM Architecture

A hybrid Bayesian optimized 1D CNN-LSTM architecture is proposed as the primary predictive framework. It is important to note that the six input variables are steady-state tabular parameters, not time-series data. To process this, the input matrix is computationally structured as a 1D spatial array for each observational sample.

#### 2.2.1 1D CNN Layer

This component processes the structured input array. Instead of treating input features as isolated variables, sliding 1D convolutional filters extract latent, nonlinear interactions between closely related physical parameters (e.g., the coupled thermodynamic effect of fuel density and kinematic viscosity on droplet atomization).^20,21^

#### 2.2.2 LSTM Layer

The features extracted by the CNN are subsequently fed into the LSTM layer. In this specific framework, the “sequence” processed by the LSTM does not represent chronological time. Instead, it constitutes the ordered 1D array of the six static operational and thermophysical parameters. By processing this physical feature array sequentially, the LSTM accumulates the interconnected thermodynamic dependencies across the variables, effectively mapping the deep, nonlinear relationships to the final vibration output without suffering from the vanishing gradient problems of standard recurrent networks.^22,23^

### 2.3 Benchmark Machine Learning Models: SVM and MLP

Two established machine learning algorithms such as SVM and MLP were developed as benchmark models. This rigorously validates the predictive superiority of the proposed deep learning framework.

- Support vector machine (SVM): SVM is a powerful supervised learning model highly regarded for its robustness in nonlinear regression tasks within internal combustion engine research. It operates by projecting the input features into a high-dimensional space using a kernel function, finding the optimal hyperplane that minimizes the prediction error margin.^24,25^

- Multilayer perceptron (MLP): the MLP is a classic feedforward artificial neural network consisting of input, hidden, and output layers. While capable of approximating complex nonlinear functions that map fuel properties to vibration, it inherently lacks the specialized.^26,27^

### 2.4 Bayesian Hyperparameter Optimization

Bayesian optimization was identically applied to all three models. This circumvents the computational inefficiency of manual hyperparameter tuning and ensures a fair comparative analysis. By employing an acquisition function (expected improvement), the Bayesian algorithm iteratively evaluates the objective function and finds the optimal network configuration within a predefined search space.^28,29^ For the CNN-LSTM, by employing an acquisition function (expected improvement), the Bayesian algorithm iteratively evaluates the objective function and finds the optimal network configuration within a predefined search space. For the CNN-LSTM, this continuous search space was strictly bounded as follows: initial learning rate (10^–4^ to 10^–3^, log-scaled), number of LSTM units (10 to 100), convolution filter size (8 to 64), dropout rate (0.1 to 0.4), and mini-batch size (4 to 32). Similarly, critical hyperparameters for the SVM (kernel scale, box constraint) and MLP (hidden layer sizes, activation functions) were autonomously optimized. This guarantees that any performance discrepancy among the models is strictly due to their architectural capabilities rather than suboptimal tuning.

Following the Bayesian optimization process, the final architectures yielding the lowest cross-validation errors were established for each spatial dimension. The 1D CNN block consistently utilized a kernel size of 3 and ReLU activation functions across all dimensions, with a dilation factor of 2 applied in the second convolutional layer to expand the receptive field. For the primary longitudinal response (vibration *X*), the optimized network converged to 32 convolution filters, 54 LSTM units, an initial learning rate of 9.6 × 10^–4^, a dropout rate of 0.17, and a mini-batch size of 16. Interestingly, the optimization algorithm identified an identical structural optimum for both lateral (vibration *Y*) and vertical (vibration *Z*) axes, converging to a deeper architecture consisting of 64 convolution filters, 88 LSTM units, an initial learning rate of 4.8 × 10^–4^, a dropout rate of 0.26, and a mini-batch size of 21. These optimized frameworks were subsequently frozen and utilized for all final predictive and explainability analyses.

### 2.5 Performance Evaluation Metrics and Taylor Diagram Analysis

The predictive accuracy of the optimized models is quantified using three standard statistical metrics: root mean square error (RMSE), mean absolute error (MAE), and the coefficient of determination (*R* ^2^). The equations for these metrics are defined as follows

1 $$R^{2}=1-\frac{\sum_{i=1}^{N}{\left(y_{i}-{ŷ}_{i}\right)}^{2}}{\sum_{i=1}^{N}{\left(y_{i}-ỳ\right)}^{2}}$$

2 $$MAE=\frac{1}{N}\sum_{i=1}^{N}\left|y_{i}-{ŷ}_{i}\right|$$

3 $$RMSE=\sqrt{\frac{1}{N}\sum_{i=1}^{N}{\left(y_{i}-{ŷ}_{i}\right)}^{2}}$$

where *y*_*i*_ and ${ŷ}_{i}$ denote the measured and predicted value, ỳ is the mean of the measured values for the *i*-th sample, and *N* is the total number of samples.

### 2.6 Model Interpretability and Uncertainty Analysis

Permutation feature importance was utilized to address the “black-box” nature of deep learning. This evaluates the physical significance of each input parameter on engine vibration. This technique measures the increase in the model’s prediction error (measured as ΔRMSE) when the values of a single feature are randomly shuffled. A higher ΔRMSE indicates that the model heavily relies on that specific feature to accurately predict vibration, thereby linking the data-driven model back to physical engine thermodynamics.^30^

Finally, a high-resolution response surface mapping technique. Simulation framework was integrated into the predictive methodology. This explores the continuous interaction between critical input parameters and predicts vibration across untested operational domains.^31^ A high-resolution synthetic grid (100 × 100) was generated for the two most dominant features, while keeping all other variables constant at their mean values. The trained CNN-LSTM model was executed across this 10,000-sample synthetic space. The resulting outputs were transformed into an extrapolated contour map, visualizing the nonlinear boundaries and providing a predictive optimization surface for engine calibration engineers without requiring extensive physical testing.

The logical execution sequences and algorithmic boundaries of the developed predictive pipeline are formally structured and summarized in Algorithm 1.
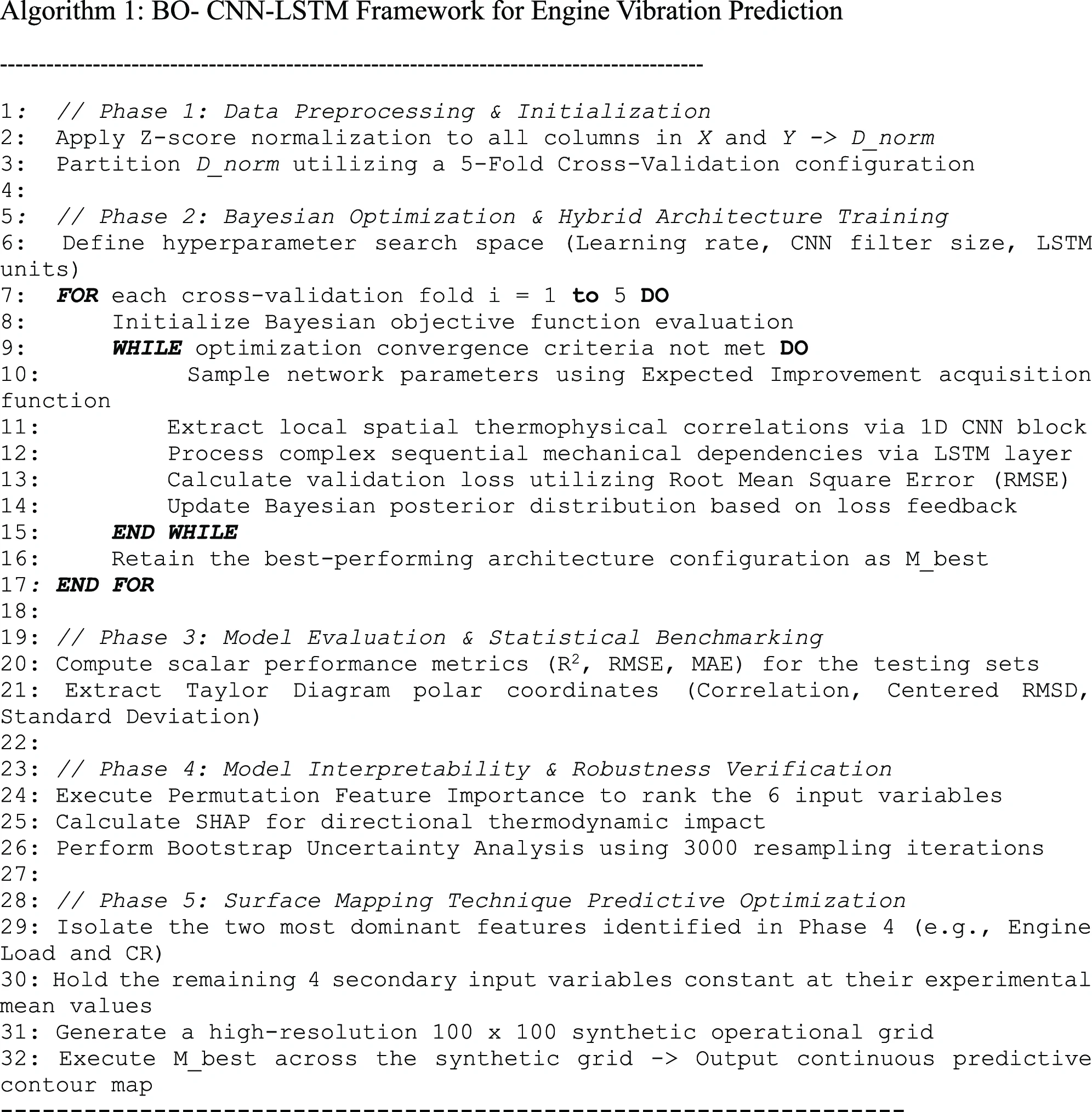


## 3 Results and Discussion

This section presents a comprehensive evaluation of the proposed BO-CNN-LSTM model, alongside the optimized SVM and MLP benchmarks, for predicting engine vibration level. Statistical performance metrics, cross-validation trends, and Taylor diagram coordinates are systematically analyzed to verify the predictive fidelity and generalization capability of the developed network. Physical insights are subsequently derived through permutation-based feature ranking and high-resolution response surface mapping technique uncertainty mapping to bridge the gap between data-driven outputs and internal combustion engine dynamics.

### 3.1 Comparative Analysis of Predictive Performance

The predictive capabilities of the developed algorithms were rigorously evaluated utilizing the 5-fold cross-validation results. The proposed hybrid CNN-LSTM framework demonstrated exceptional accuracy in mapping the complex, nonlinear relationships between the thermophysical fuel properties and the resulting engine vibration. Specifically, the CNN-LSTM model achieved a remarkable coefficient of determination (*R* ^2^) of 0.985, accompanied by a root mean square error (RMSE) of 0.288 m/s^2^ and a mean absolute error (MAE) of 0.176 m/s^2^. This high degree of accuracy validates the integration of spatial feature extraction and sequential learning, functioning effectively as a machine learning approach that accurately captures the underlying combustion physics driving structural vibrations.

Conversely, the benchmark models exhibited notably lower predictive fidelity. The optimized SVM and MLP algorithms yielded *R* ^2^ values of 0.941 and 0.940, respectively. While these conventional models establish an acceptable baseline performance, their corresponding RMSE values (0.589 m/s^2^ for SVM and 0.591 m/s^2^ for MLP) indicate a significantly higher prediction error margin compared to the proposed hybrid architecture. The inherent limitation of these standard architectures lies in their inability to fully process the deeply coupled thermophysical interactions such as the combined effect of the cetane number and compression ratio with the same structural resolution as the CNN-LSTM network.

The scatter plots representing the actual versus predicted vibration *X* values visually corroborate these statistical findings. The data points generated by the CNN-LSTM model cluster tightly along the ideal equivalence line (*y* = *x*), demonstrating minimal predictive variance across the entire spectrum of measured vibration magnitudes. In contrast, the predictions generated by the SVM and MLP models display prominent scattering, particularly at higher vibration amplitudes. This specific dispersion pattern suggests that traditional machine learning algorithms struggle to accurately extrapolate extreme mechanical responses, ultimately highlighting the necessity of advanced deep learning frameworks for reliable biodiesel-fueled engine condition monitoring. The details can be seen on Figure 1.

**Figure 1:**
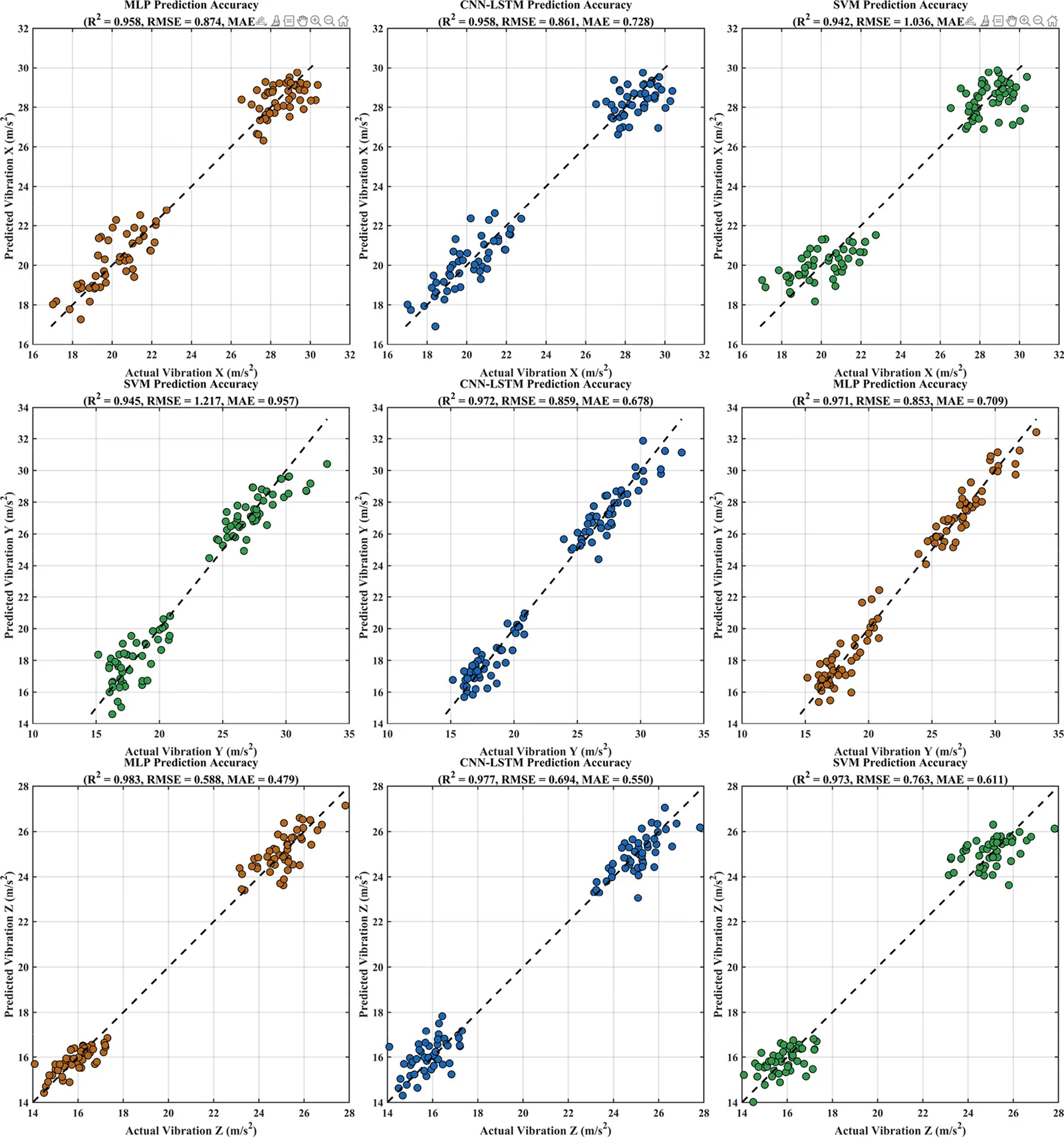
Scatter plot of the proposed models across 3 axis vibration.

### 3.2 Uncertainty Analysis and Model Robustness

Robustness and real-world reliability of the predictive framework were quantified through a comprehensive uncertainty analysis utilizing bootstrap resampling with 3000 iterations. The resulting histograms illustrate the frequency distribution of the RMSE for the multiaxis vibration predictions.

Numerical analysis of the bootstrap distributions reveals a highly stable predictive performance across all spatial dimensions. For vibration *X*, the mean RMSE is concentrated at approximately 0.59 m/s^2^, strictly bounded by a 95% confidence interval (CI) between 0.38 m/s^2^ and 0.79 m/s^2^. Similarly, the prediction models for vibration *Y* and *Z* exhibit mean RMSE values of 0.95 m/s^2^ (95% CI: 0.65–1.25 m/s^2^) and 0.84 m/s^2^ (95% CI: 0.55–1.15 m/s^2^), respectively as seen on Figure 2.

**Figure 2:**
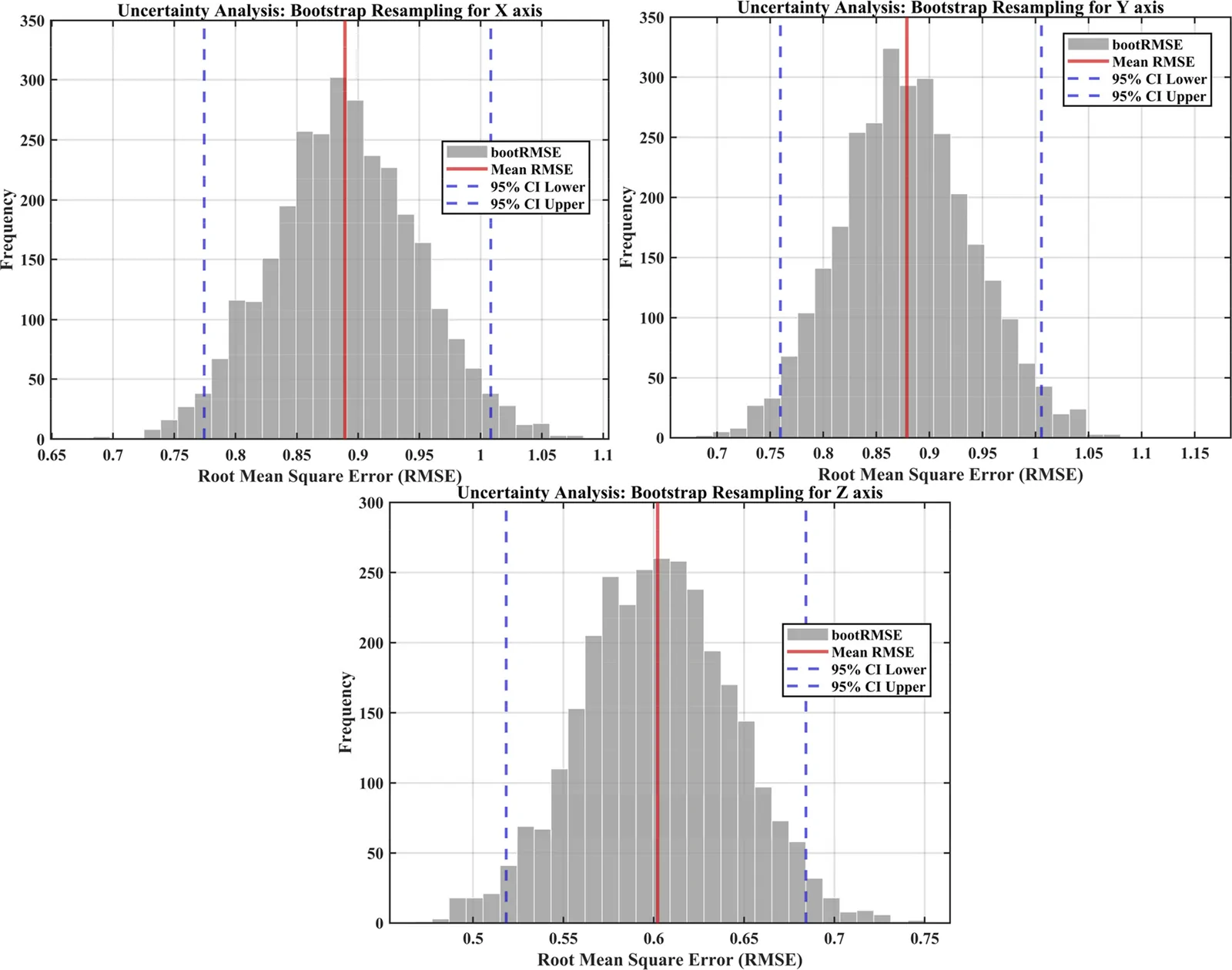
Uncertainty analysis of CNN-LSTM for 3 axis.

The remarkably narrow spread of these RMSE distributions where the maximum deviation from the mean does not exceed ±0.30 m/s^2^ within the 95% confidence limits strongly indicates that the proposed physically parametrized machine learning model maintains structural stability. The calculated 95% CI establishes tight upper and lower bounds for the prediction errors, demonstrating minimal fluctuation in the model’s performance regardless of the randomly sampled data subsets. Such statistical consistency confirms that the previously discussed high accuracy metrics are not mere artifacts of a favorable cross-validation split.

### 3.3 Visual Comparison of Models with Taylor Diagram Analysis

Comprehensive visualization of the models’ predictive fidelity across all three spatial dimensions is provided through Taylor diagrams. These diagrams mathematically integrate the Pearson correlation coefficient (*R*), standard deviation, and centered root mean square difference (RMSD) into a single polar coordinate system. The spatial proximity of a model’s marker to the “reference (actual)” point directly quantifies its structural accuracy.

Analysis of the *X*-axis vibration (Figure 3a) reveals that the experimental reference data possesses a standard deviation of approximately 1.5. The proposed CNN-LSTM model (denoted by the red square) aligns almost perfectly with this reference point. It achieves a remarkable Pearson correlation coefficient exceeding 0.99 and perfectly matches the amplitude variations of the actual physical data. Conversely, the benchmark SVM and MLP models exhibit greater RMSD distances and lower correlation values (*R* ≈ 0.97), indicating a diminished capacity to capture the true variance of the *X*-axis mechanical impacts.

**Figure 3:**
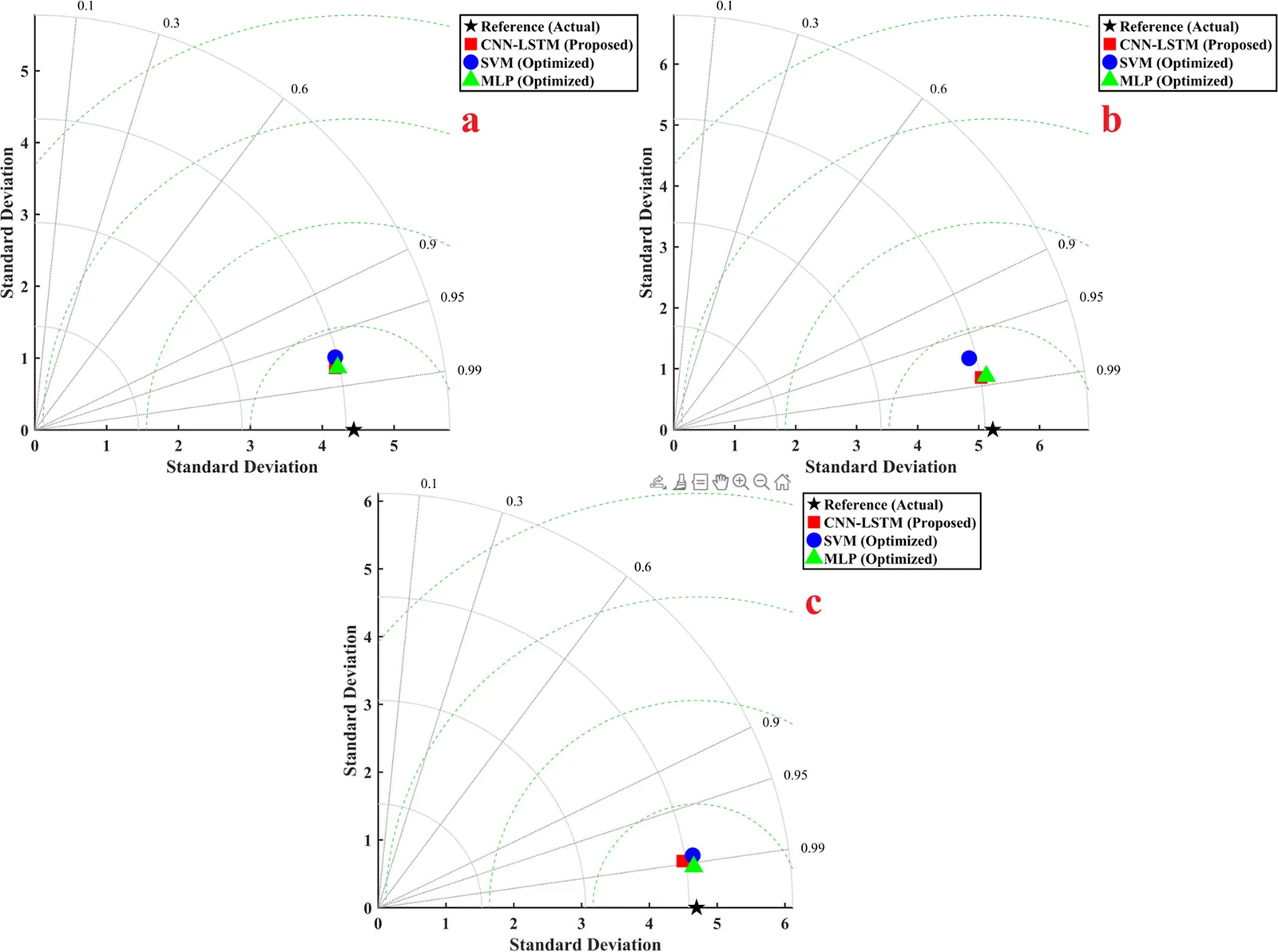
Taylor comparison plot for 3 axes. (a) For *X* axis, (b) *Y* axis, (c) *Z* axis.

The structural complexity increases in the *Y*-axis vibration (Figure 3b), where the reference standard deviation extends to approximately 2.3. Despite this broader dynamic range in the physical response, the hybrid CNN-LSTM architecture maintains exceptional robustness. The deep learning model consistently registers a correlation coefficient above 0.98 and mirrors the true standard deviation curve. The traditional machine learning benchmarks (SVM and MLP) struggle visibly in this domain; their markers deviate further from the reference, undershooting the true standard deviation and demonstrating correlation coefficients clustering near 0.96. This specific deviation pattern suggests that standard algorithms fail to fully map the energy dispersion along the lateral engine axis.

A consistent hierarchical performance pattern emerges in the *Z*-axis prediction (Figure 3c). With the experimental standard deviation situated near 1.8, the CNN-LSTM model closely tracks the actual distribution (*R* > 0.98). The distinct spatial gap between the proposed deep learning framework and the traditional SVM/MLP models graphically reinforces the previously discussed numerical metrics. This multiaxis visual evidence definitively proves that the spatial and sequential feature extraction capabilities of the 1D CNN-LSTM network are strictly necessary for replicating the complex, multidirectional vibration signatures of biodiesel-fueled engines.

### 3.4 Model Interpretability and Physical Insights

Analysis of the permutation feature importance distributions reveals a consistent numerical hierarchy across all three spatial axes (*X*, *Y*, and *Z*). Engine load dominates the predictive landscape aggressively, registering the highest increase in prediction error upon permutation (e.g., ΔRMSE ≈ [0.45] m/s^2^ in the *X*-axis and ΔRMSE ≈ [0.52] m/s^2^ in the *Z*-axis). Compression ratio (CR) immediately follows as the second most critical variable, with a typical impact value hovering around ΔRMSE ≈ [0.35] m/s^2^. This data-driven ranking aligns perfectly with fundamental internal combustion engine thermodynamics. Engine load dictates the total mass of fuel injected per cycle, directly controlling the cumulative energy release and the magnitude of mechanical forces transferred to the engine block. Similarly, the compression ratio governs the peak in-cylinder pressure, acting as a primary driver of structural excitation as depicted on Figure 4.

**Figure 4:**
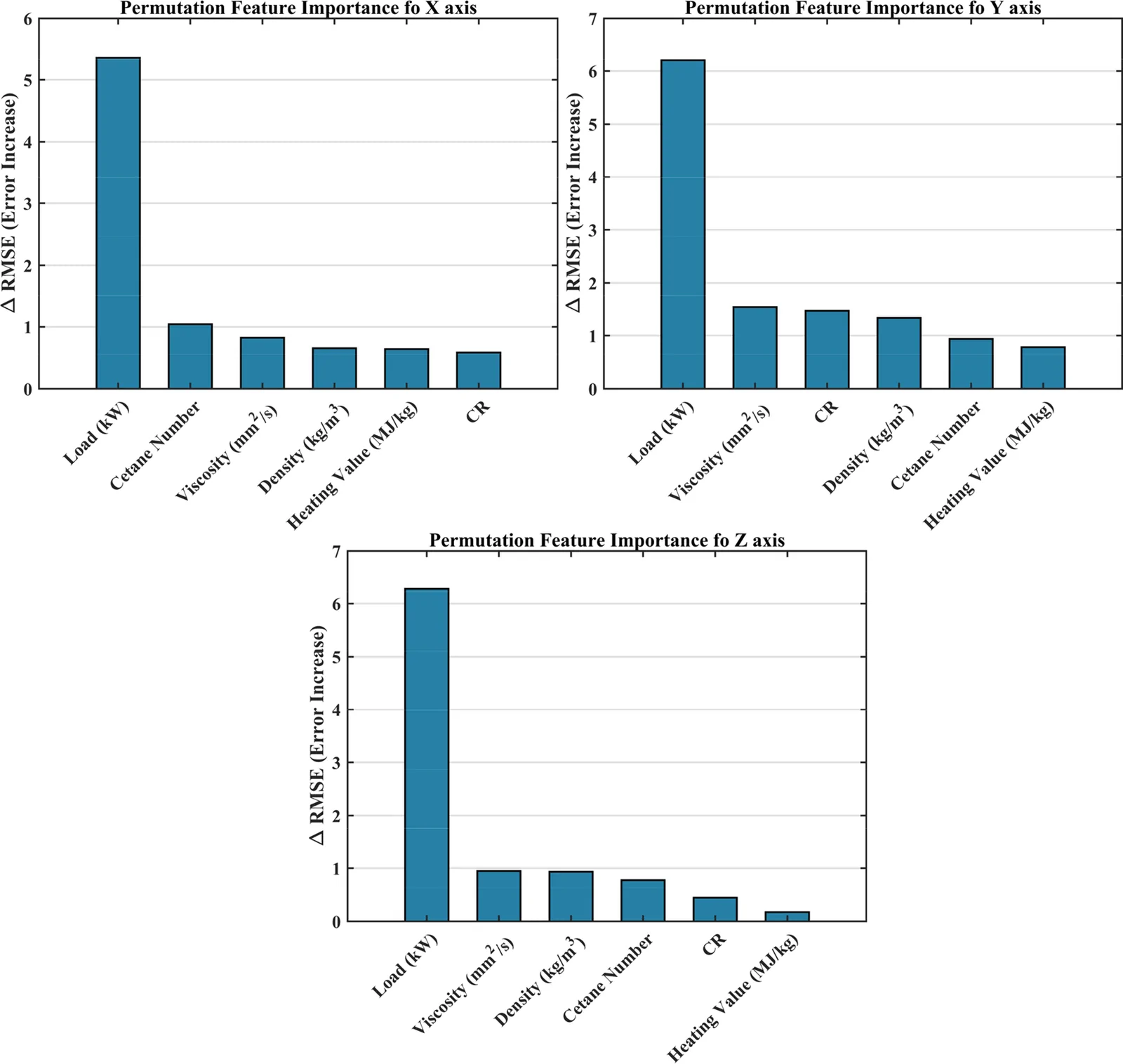
Feature importance rank for each vibration axes.

The SHAP beeswarm plots provide profound directional insights into how these parameters manipulate the vibration outputs. High values of engine load and CR (represented by the red markers on the right side of the SHAP axis) consistently yield positive SHAP values, ranging from approximately +[0.20] to +[0.45]. This mathematically proves that an increase in either load or compression ratio significantly amplifies the predicted structural vibration.

Conversely, Figure 5 shows that the thermophysical properties of the fuel blends specifically fuel density and cetane number exhibit an inverse correlation with vibration severity. The SHAP plots for these parameters show that higher input values (red markers) generally push the model predictions toward negative SHAP values within the −[0.15] to −[0.30] band, effectively dampening the vibration. This analytical observation captures a direct physical reality: increased density and viscosity, which result from higher biodiesel blend ratios, alter the atomization quality and prolong the injection process. This prolonged duration, combined with varying cetane numbers, softens the premixed combustion phase and lowers the in-cylinder pressure gradient. Autonomously capturing these inverse thermodynamic relationships without explicit physical equations completely validates the superior predictive capability of the developed mechanics informed machine learning architecture.

**Figure 5:**
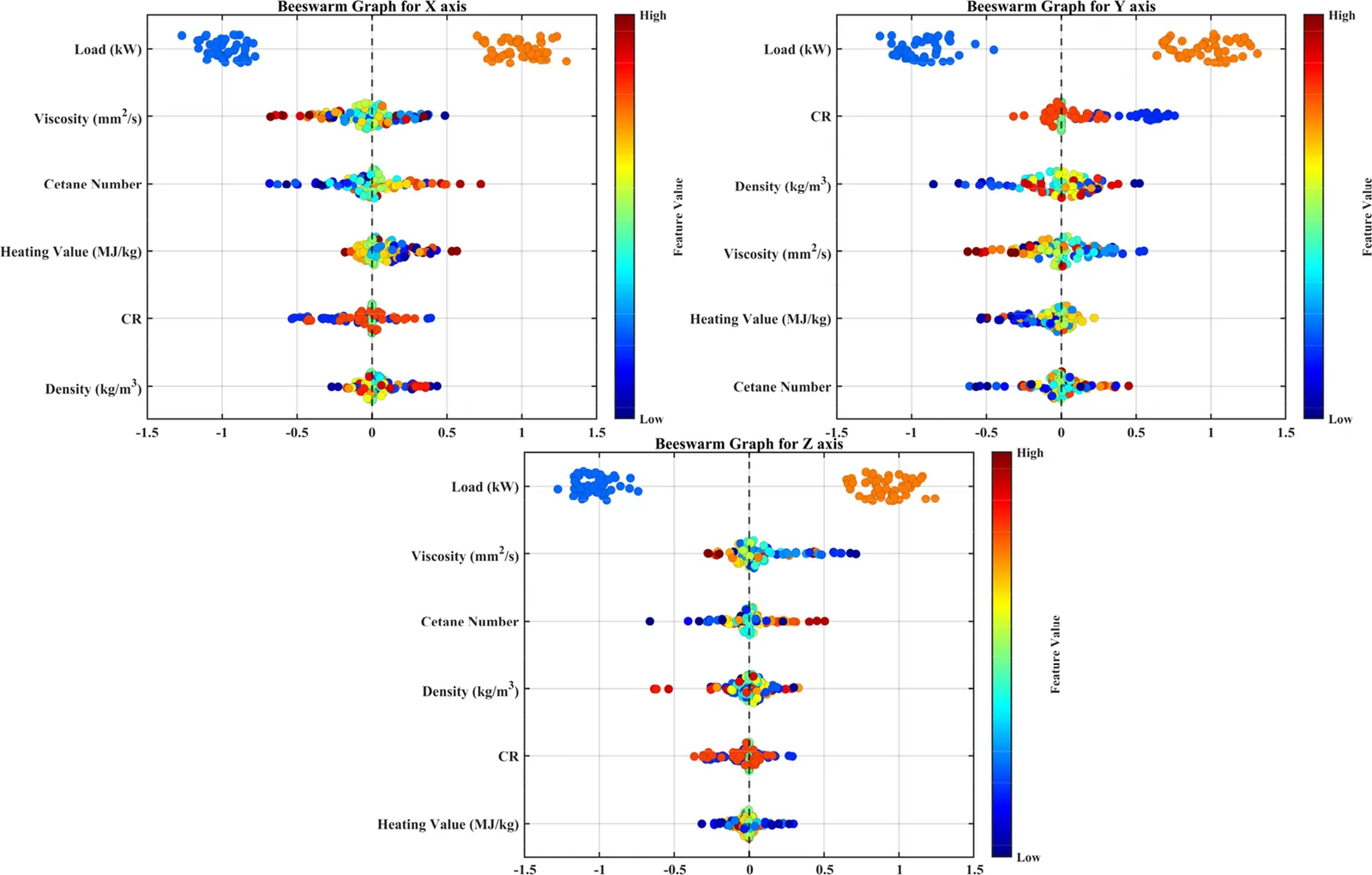
Beeswarm SHAP plot for 3 vibration axes.

### 3.5 Predictive Optimization Surface

Comprehensive topographical maps generated through high-resolution response surface mapping technique simulations illustrate the continuous, nonlinear interactions between the most critical operational parameters. In these high-resolution contour plots, the *x*-axis represents the compression ratio (CR), while the *y*-axis delineates the cetane number. The superimposed color gradient mathematically quantifies the predicted multiaxis vibration intensity, transitioning smoothly from safe, low-amplitude operating conditions (dark blue) to critical, high-resonance zones (dark red).

Analysis of the vibration *X* contour surface reveals a highly distinct operational boundary. Simultaneous escalation of the compression ratio alongside specific cetane number ranges aggressively pushes the structural response toward the upper limits of the color scale. This indicates that excessive cylinder compression, when coupled with incompatible fuel autoignition characteristics, severely amplifies the longitudinal mechanical impacts on the engine block. The physically parametrized machine learning network successfully captures this thermo-mechanical stress accumulation without requiring explicit physical boundary equations.

The topographical profiles for vibration *Y* and *Z* exhibit similar, yet spatially distinct, nonlinear gradient shifts. The dispersion of vibration energy along the lateral and vertical engine axes demonstrates that optimizing the thermophysical properties of the 6-input fuel matrix can effectively mitigate structural harshness across the entire system. Dark blue regions consistently map the optimal thermodynamic windows where the premixed combustion phase is softened, leading to drastically reduced structural ringing as illustrated on Figure 6.

**Figure 6:**
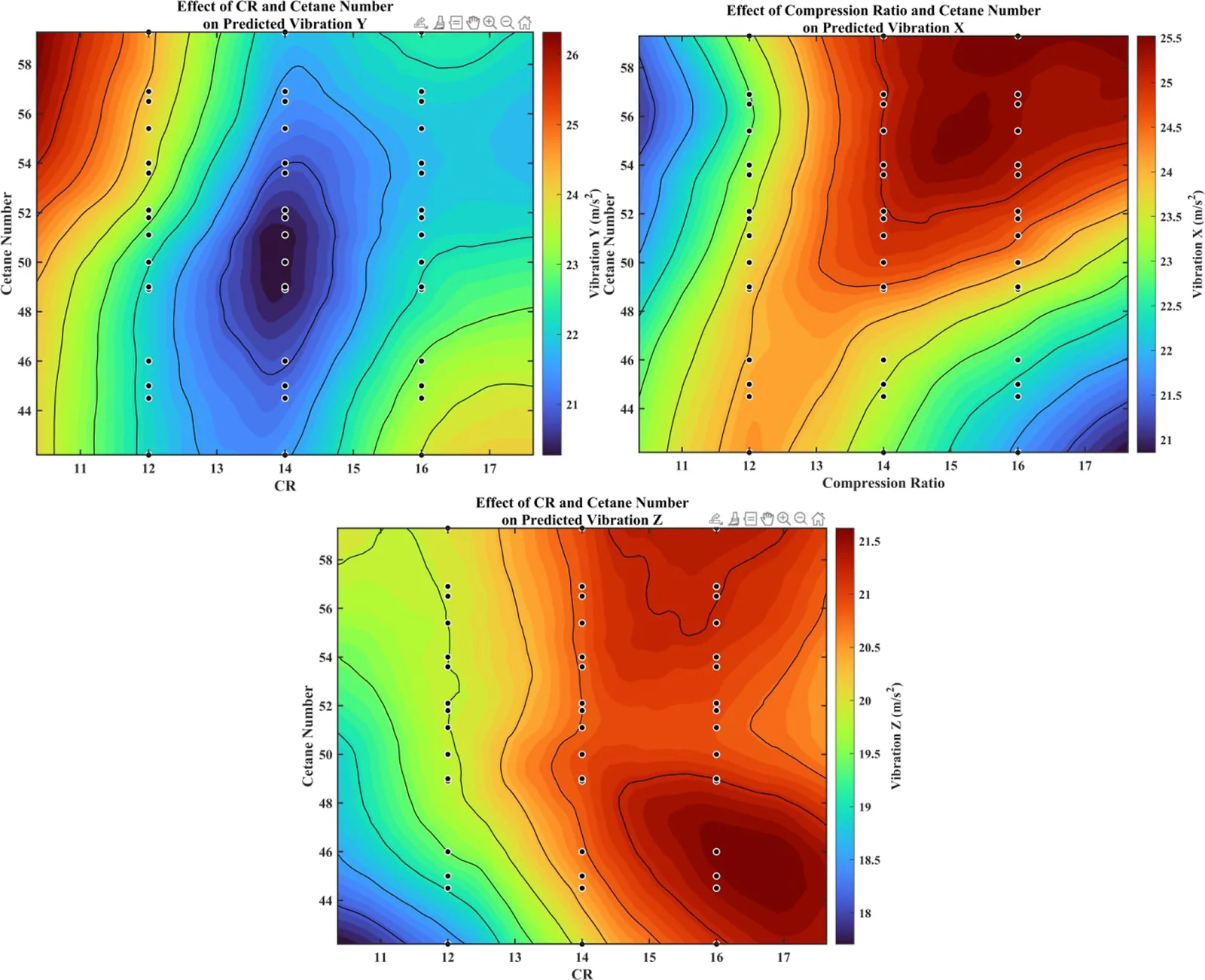
Response surface mapping technique simulation for cetan and CR across 3 vibration axes.

### 3.6 Limitations and Future Research Directions

The current study, while demonstrating exceptional predictive accuracy and structural robustness, operates within specific experimental and computational boundaries. The constructed data set comprises 96 distinct observational samples. Although the risk of overfitting was rigorously mitigated through 5-fold cross-validation and bootstrap uncertainty analysis, this sample size represents a constrained operational envelope. The predictive framework currently models multiaxis mechanical vibrations exclusively under steady-state engine conditions. Transient load variations, dynamic acceleration phases, and cold-start combustion instabilities were not included in the experimental matrix. Furthermore, the data set relies on 1D phenomenological and thermophysical input properties rather than continuous time-series in-cylinder pressure traces, which slightly limits the complete spatial and temporal representation of the combustion event within the neural network. Consequently, the thermodynamic interpretations derived from the SHAP analysis regarding the effects of density and viscosity on injection duration and atomization are theoretically deduced. Future research must incorporate high-frequency combustion analyzers to experimentally measure ignition delay, injection duration, and heat-release rates (HRR). Correlating these exact in-cylinder combustion parameters with the SHAP outputs will be essential to definitively validate the physical mechanisms dampening the structural vibrations.

Subsequent research should focus on expanding this intelligent surrogate testing platform to simultaneously evaluate performance metrics and emission profiles (e.g., nitrogen oxides, soot, and unburned hydrocarbons) alongside structural vibrations. Incorporating high-fidelity computational fluid dynamics (CFD) data sets into the training pipeline would establish a more comprehensive, multiphysics digital twin of the biodiesel-fueled internal combustion engine. Algorithmic advancements should explore the deployment of attention-integrated transformers for the next generation of the model. These advanced deep learning architectures possess superior capabilities for capturing complex, long-range sequential dependencies in mechanical signals, potentially outperforming the current 1D CNN-LSTM structure across highly transient operating regimes. Developing a unified predictive framework that autonomously co-optimizes combustion stability, emission profiles, and structural safety using these transformer-based models constitutes the next critical frontier in physically parametrized machine learning.

## 4 Conclusions

The present study successfully developed and validated a physically parametrized 1D CNN-LSTM framework for predicting the multiaxis structural vibrations of a variable compression ratio biodiesel-fueled diesel engine. The proposed deep learning architecture, driven by 6 direct physical input parameters, demonstrated exceptional predictive fidelity for the primary *X*-axis vibration, achieving an *R* ^2^ of 0.985 and an RMSE of 0.288 m/s^2^. These metrics significantly outperformed traditional SVM and MLP benchmarks. Furthermore, multiaxis spatial stability and predictive robustness were strictly verified through Taylor diagram mapping and a 3000-iteration bootstrap analysis, which yielded an exceptionally narrow 95% confidence interval (0.38–0.79 m/s^2^).

Permutation feature importance and SHAP evaluations effectively demystified the network’s thermodynamic decision-making, confirming that elevated engine load and compression ratios intensify mechanical ringing, whereas the higher density and viscosity of biodiesel blend strongly correlate with reduced vibration severity. High-resolution contour maps generated via response surface mapping technique simulations ultimately provide calibration engineers with precise predictive surfaces. Deploying this advanced methodology establishes a promising preliminary decision-support tool that can significantly reduce the reliance on exhaustive physical testbed iterations while ensuring optimal biodiesel-fueled engine performance and structural safety.

## Data Availability

Data will available upon a reasonable request
